# Evaluating Collective Impact for Healthy Aging at the Intersection of Public Health and Age-Friendly Communities

**DOI:** 10.1093/geroni/igaa057.2450

**Published:** 2020-12-16

**Authors:** Kathy Black, Kathryn Hyer

**Affiliations:** 1 University of South Florida, Sarasota-Manatee, Sarasota, Florida, United States; 2 University of South Florida, Tampa, Florida, United States

## Abstract

There is mounting interest in promoting - and evaluating efforts that improve healthy aging in age-friendly communities. Additionally, there is increasing recognition that multi-sectoral engagement beyond the aging network is needed to maximize impact and sustainability. Within the context of collective impact, this paper reviews a framework that explicates public health activities in collaboration across a range of stakeholders in age-friendly communities. Metrics demonstrating evidence of five categorical roles, processes and outcomes will be presented including: 1) Connecting and Convening; 2) Coordinating; 3) Collecting and Disseminating Data; 4) Communicating; and 5) Complementing and Supplementing. Examples that illustrate evidence vis-à-vis the components and phases of collective impact will be presented.

